# The five keys for a successful implementation of a cardiac telerehabilitation: a step-by-step effective digitalization of rehabilitation health services in Egypt

**DOI:** 10.1186/s43044-022-00324-3

**Published:** 2022-12-27

**Authors:** Hady Atef, Marwa Gaber, Bassem Zarif

**Affiliations:** 1 Department of Physical Therapy for Cardiovascular/Respiratory Disorders and Geriatrics, Faculty of Physical Therapy, Cairo University, Giza, Egypt; 2 Medical Research Institute, Alexandria University, Alexandria, Egypt; 3 National Heart Institute, Giza, Egypt

**Keywords:** Cardiac rehabilitation, Telerehabilitation, COVID era, Cardiovascular disease, Virtual rehabilitation

## Abstract

**Background:**

Telerehabilitation enables patients to communicate with physicians through the Internet and may be utilized to evaluate patients’ conditions and offer treatment plans. This method became necessary as a result of the COVID-19 pandemic and its influence on face-to-face rehabilitation choices. Many rehabilitation professionals throughout the world have turned to the ‘online’ approach, relying on smartphone and smartwatch services such as WhatsApp, Facebook, and various mobile applications that comply with the ESC requirements.

**Main body:**

Throughout this editorial, we examine the function of cardiac telerehabilitation in light of the journalistic ‘5 W,’ taking into consideration the rising interest in this topic during the ‘COVID era.’

**Conclusions:**

Telerehabilitation is the future of rehabilitation, particularly in the COVID age. Additionally, telerehabilitation has proved to be successful in the cardiac profession when compared to face-to-face treatments, implying that this type of rehabilitation may continue after the world is COVID-free, and forecasting that it would be the preferable choice in the future.

**Graphical Abstract:**

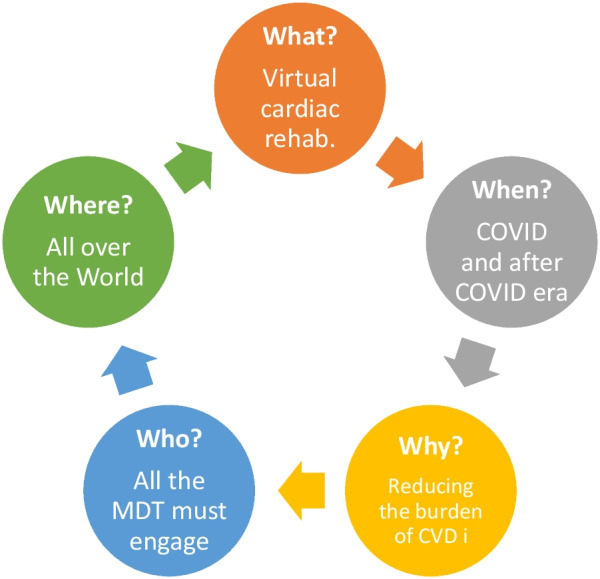

## Highlights

1. Cardiac telerehabilitation might promote the patient’s functional recovery in a safe manner, as there is no direct contact between the clinician and the patient.
2. Cardiac telerehabilitation may improve social functioning and psychological well-being by avoiding isolation.
3. The diffusion of technologies as an effective therapeutic alternative approach during the pandemic will be possible in the near future if the factors that facilitate human–technology interaction will be identified and well defined.
4. The intervention must be designed according to the user’s needs, in order to encourage acceptance of the innovative and highly technological healthcare program.
5. It is important to actively train healthcare professionals, face concerns about the remote treatment modality, and engage them to facilitate a more effective planning intervention.

## Background

Telerehabilitation (also known as e-rehabilitation) is the provision of rehabilitation services using telecommunication systems and the Internet. The European Society of Cardiology (ESC) has advocated telerehabilitation over traditional rehabilitation procedures since it has the same effectiveness and maintains COVID prevention strategies [1, 2].

COVID-19 patients often appear with respiratory tract infection symptoms, although cardiac manifestations, including evidence of myocardial damage, are not uncommon. Based on research undertaken during the earlier SARS and MERS outbreaks, as well as the continuing COVID-19 pandemic, many causes for cardiac injury have been proposed. The production of large quantities of cytokines (cytokine release syndrome) as part of the systemic inflammatory response in severe COVID-19 can harm many structures, including vascular endothelium and cardiac myocytes [3].

This medical emergency and the resulting modification of health centers had a negative impact on the rehabilitative therapies of non-COVID illnesses, adversely affecting the quality of life of patients (particularly those with cognitive diseases) and their families. In our perspective, cardiovascular function is a vital activity that must be incorporated in the rehabilitation regimens of COVID and non-COVID cardiovascular patients [4].

In this context, telerehabilitation could aid in meeting both the physical and psychosocial needs of all patients, regardless of their geographical location. It has recently been demonstrated that the application of telerehabilitation results in clinical gains that are as good as the traditional face-to-face rehabilitation programs. This is especially critical during a COVID-19 pandemic, when managing cardiovascular illnesses in adults and children is a major problem for health systems throughout the world. In this editorial, we examine the function of cardiac telerehabilitation in light of the journalistic ‘5 W,’ taking into consideration the rising interest in this topic during the ‘COVID era.’

## Main text

### WHAT?

**Fig. 1:**
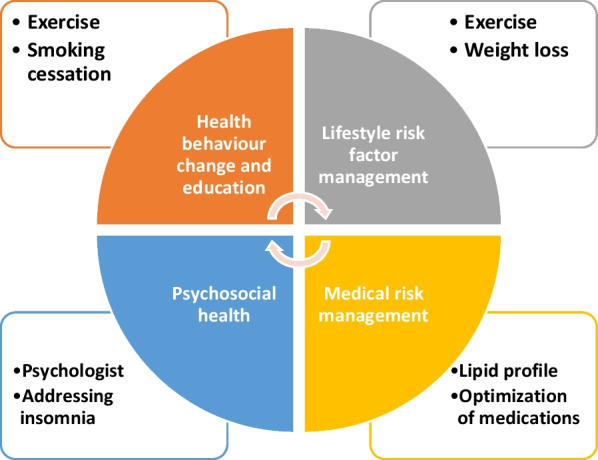
Components of an efficient cardiac rehabilitation program

Telerehabilitation is a novel and innovative method of providing meaningful assistance throughout the home rehabilitation procedure for the treatment of cardiovascular, nutritional, and psychological diseases. It can involve physiotherapy, dietary counseling, smoking reduction, patient telemonitoring, occupational therapy, and teleconsultation by home-bound patients. They are assisted by therapists or other healthcare experts who are not physically present [5] (Fig. 1).

This method entails the rehabilitation and/or compensation of reduced cardiovascular and psychological fitness, with the goal of improving the patient’s quality of life, family support, and societal environment. Cardiac telerehabilitation necessitates extensive home-based training under the guidance of a therapist through the Internet. It gives individuals who reside in remote locations or cannot have access to local healthcare providers owing to physical limitations or restrictions (in pandemics, for example) an equal chance to get rehabilitation therapies [6]. Various software are utilized for cardiac telerehabilitation, employing either the mobile phone with particular health applications or pc-based workouts, with multiple exercises including the main physical domains. These devices are designed to adjust the level of challenge of the exercises depending on the patient’s progress, as well as the ability to select different sets of exercises according to the patient’s cardiovascular state. These features are required to build activities that are suited to the patient’s condition. Platforms are designed specifically for cardiac rehabilitation patients, taking into account their needs and characteristics as outlined by cardiac rehabilitation physicians and psychologists and engineers [7]. The platforms feature unique tracking systems that enable video conferencing and safe electronic data transmission. Clinical examinations and monitoring of patients’ cardiovascular workouts can be performed by healthcare experts. Patients can contact physicians and resolve self-training and/or technical issues by exchanging voice messages/e-mails or by participating in a video call. Moreover, home cardiac telerehabilitation may necessitate rigorous and daily treatments, and the customization of the intervention provides for constant feedback to the patient, who becomes more engaged as a result of a higher knowledge of their physical capabilities [8].

Several studies have demonstrated that cardiac telerehabilitation is effective, simple to use, motivating, and available to a wide range of patients; therefore, it may be valuable in relieving the present strain on the healthcare system caused by the pandemic [9].

### WHEN?

COVID-19 produced several changes in people’s lifestyles, forcing them to adapt their everyday routines and activities. These alterations primarily influence the subject’s physical and behavioral spheres, potentially affecting quality of life, particularly in frail individuals [8]. In principle, cardiac rehabilitation is seen as required for individuals suffering from cardiovascular illness to prevent long-term heart damage and mitigate the related social and psychological repercussions. It may be tailored to both the COVID-19 positive group to minimize the cardiovascular sequelae seen after hospitalization and psychiatric patients to restrict and improve the prognosis of cardiovascular impairments [10].

The success of cardiac rehabilitation treatment rises when the stimulation sessions are vigorous and constant, even while the patient is at home [1].

Cognitive function stimulation in the presence of a health emergency, such as COVID-19, uses the possibility of remote telerehabilitation to enhance cardiovascular fitness, independent of the diagnosis (congenital or acquired impairment), the participants’ cardiovascular profile, or the patient’s age (developmental, adult, or senior). This enables the provision of various activities that include and encourage patients to improve their quality of life [7]. People can stay at home comfortably without compromising cardiovascular fitness and avoiding such a severe sickness. This new strategy might be employed not just during the pandemic, but also when the ‘lockdown’ time ends, implying that we can apply the ‘hybrid’ method even after the COVID era ends.

### WHY?

National health systems are constantly developing via restructuring of primary care, integrating diverse levels of care, and continuity of treatment. Cardiac telerehabilitation can be used to provide rehabilitation services when the healthcare provider and the patient (or both) are not in the same area [10]. Conventional cardiac rehabilitation has a few limits in terms of time, expense, and patient accessibility, particularly given the ‘restrictions’ imposed for the pandemic. The application of rehabilitation technology enables the delivery of services virtually, in a ‘non-medical’ context, with innovative rehabilitation programs that expand the potential and consistency of treatment. During critical times such as the COVID-19 emergency, cardiac telerehabilitation via ICT (Information and Communication Technology) can provide care continuity and exact assessment of patient performance. ICT provides precise and trustworthy patient management by impacting attitudes and behaviors toward everyday life and possibly enhancing their mental and medical constraints, reducing the overload on healthcare workers who are occupied in the struggle against COVID-19 and unable to take the necessary measures in the emergency situations unassociated with the pandemic [2].

### WHO?

For the application of cardiac telerehabilitation, two types of patients must be taken into account: users and operators (mainly clinicians). The healthcare professional plays a critical role in including patients in the treatment process. Accurate cardio-psychological evaluation determines cardiovascular/psychological treatment, including customization, physical exercise settings and feedback, and patient engagement. As a result, it is critical to teach clinicians working in ICT to satisfy the many demands that this period and the new forms of help need. Furthermore, effective operator training can stimulate the usage of these novel technologies, alleviating worries about the cost and time required for employing tele-rehab services. Previously, platforms needed licenses and limited fees, which are now being addressed by the emergence of new low-cost technologies (such as Skype and Android applications) that enable the delivery of professional services while lowering the expenses associated with cardiac telerehabilitation [5].

Telerehabilitation can help all individuals who have a cardiovascular deficiency. Previous research has emphasized the system’s applicability and usefulness for both developmental and adult cardiovascular problems, as well as geriatric patients [1].

Several countries throughout the world have been on lockdown since March 2020 and experience a number of issues. The main challenge currently is weighing the health benefits against the negative psychological, social, and economic effects of the lengthy lockdown. In terms of cardiovascular illnesses, the unforeseen calamity might induce or worsen a variety of physical, cognitive, and behavioral issues that can be successfully handled using ICT.

### WHERE?

All countries throughout the world can benefit from telerehabilitation, particularly the COVID-19-affected countries where rehabilitation institutions and local services have either ceased to exist or have been turned into COVID centers. This novel solution should be made available to patients residing in rural, island, and mountain locations. Moreover, cardiac telerehabilitation can eliminate the necessity for patients to visit hospital facilities, which is especially beneficial for senile patients and those with persistent multiple illnesses [8]. Telerehabilitation provides secure accessibility to rehabilitation treatments for individuals residing in rural places or regions where access to either patients or clinicians is limited owing to COVID-19 regulations. For security and clinical practice considerations, the platform and accompanying ICT services should adhere to the principles outlined in the WCPT/INPTRA Digital Practice Final Report, which was published in March 2020.

## Conclusions

We believe that cardiac telerehabilitation could play an important role in the COVID-19 era, both in terms of promoting the patient’s functional recovery in a safe way, as there is no direct contact between the clinician and the patient, and enhancing social functioning and psychological well-being by avoiding isolation. If the characteristics that promote human–technology interaction are recognized and clearly established, the dissemination of technologies as a successful therapeutic alternative method during pandemics will be achievable in the near future. To encourage adoption of the novel and highly technical healthcare program, the intervention must be developed according to the demands of the users. It is critical to actively teach health workers, address their worries regarding distant therapy, and involve them to promote a more successful planned intervention. One of the proposed applications that might be used to foster the process of telerehabilitation is the use of zoom and/or Microsoft teams meetings. The good things about these applications are that both of them are simple to use, freely available to be downloaded from either google play or the app store, and offer free usage for limited-time meetings. These features might encourage both patients and clinicians for giving telerehabilitation a try, in a way to push healthcare delivery in Egypt forward.

## Data Availability

All data generated or analyzed during this study are included in this published article.
